# Immunostimulatory properties and antitumor activities of glucans

**DOI:** 10.3892/ijo.2013.1974

**Published:** 2013-06-05

**Authors:** LUCA VANNUCCI, JIRI KRIZAN, PETR SIMA, DMITRY STAKHEEV, FABIAN CAJA, LENKA RAJSIGLOVA, VRATISLAV HORAK, MUSTAFA SAIEH

**Affiliations:** 1 Laboratory of Immunotherapy, Department of Immunology and Gnotobiology, Institute of Microbiology, Academy of Sciences of the Czech Republic, v.v.i., 142 20 Prague 4;; 2 Laboratory of Tumour Biology, Institute of Animal Physiology and Genetics, Academy of Sciences of the Czech Republic, v.v.i., 277 21 Libechov, Czech Republic;; 3 Department of Biology, University of Al-Jabal Al-Gharbi, Gharyan Campus, Libya

**Keywords:** β-glucans, polysaccharides, immunity, immunotherapy, cancer

## Abstract

New foods and natural biological modulators have recently become of scientific interest in the investigation of the value of traditional medical therapeutics. Glucans have an important part in this renewed interest. These fungal wall components are claimed to be useful for various medical purposes and they are obtained from medicinal mushrooms commonly used in traditional Oriental medicine. The immunotherapeutic properties of fungi extracts have been reported, including the enhancement of anticancer immunity responses. These properties are principally related to the stimulation of cells of the innate immune system. The discovery of specific receptors for glucans on dendritic cells (dectin-1), as well as interactions with other receptors, mainly expressed by innate immune cells (e.g., Toll-like receptors, complement receptor-3), have raised new attention toward these products as suitable therapeutic agents. We briefly review the characteristics of the glucans from mycelial walls as modulators of the immunity and their possible use as antitumor treatments.

## Contents

1. Introduction
2. β-glucan sources and properties
3. Immunostimulatory properties of glucans
4. Glucan receptors on immune cells
5. Antitumor activities of glucans
6. Conclusion and potential

## 1. Introduction

Renewed interest has recently arisen for both functional foods and the investigation of the scientific value of traditional medical treatments. The evaluation of mushroom derivatives and their medical properties are important part of these studies. Polysaccharides, including the glucans, have been described as biologically active molecules (1–4). Certain glucose polymers, such as (1→3), (1→6)-β-glucans, were recently proposed as potent immunomodulation agents (3–5). Even though glucans can be extracted from the cell walls of yeast, oat, barley, seaweeds, algae and bacteria, the foremost source of medical glucans turns out to be fungal cell walls which consist either of polysaccharides such as chitin, cellulose, (1→3)-, (1→6)-β-glucans and (1→3)-α-glucans, or polysaccharide-protein complexes (6). The β-glucans are the most studied within these polysaccharides and are principally obtained from the fruit body of various types of mushrooms. Used especially in traditional Oriental medicine (7), they are reported to be found in fruit bodies, cultured mycelium and cultured broth from higher Basidiomycetes mushrooms (as resulted from ∼700 species of investigated higher Hetero- and Homobasidiomycetes). According to traditional medicine assertions, as well as some scientific studies, glucans have been reported to produce anti-tumor, immunomodulating, antioxidant, radical scavenging, cardiovascular, antihypercholesterolemia, antiviral, antibacterial, antiparasitic, antifungal, detoxification, hepatoprotective and antidiabetic effects (8,9). Growing interest of Western science into biologically active polysaccharides can be considered to start after the publication of Pillemer and Ecker in 1941 (10). They described a crude yeast cell wall preparation, later named zymosan, able to modulate non-specific immunity (complement) (11). It was unknown at that time which component of this preparation was stimulating the immune response. Later on, β-glucan was identified by Riggi and Di Luzio as the immune-activating compound within the components of zymosan (after testing its lipid and mannan components) (7). Since then, a large number of studies have been performed to clarify the immunomodulating potential of glucans and their possible antitumor effects (12–18). The discovery of specific receptors for glucans on immune cells, the recent advances in understanding host immune responses against infectious agents and cancer and the importance of the innate immunity (inflammation) in these responses have renewed the interest toward glucans as perspective immunotherapeutic molecules.

## 2. β-glucan sources and properties

The glucans are D-glucose-based polysaccharides. With their glucose anomeric structure, they can be α-D-glucans, β-D-glucans and mixed α,β-D-glucans. They present different types of glycosidic bonds originating either (1→3)-, (1→6)-β-glucans (e.g., zymosan, laminarin, lentinan, pleuran), or (1→3)-, (1→4)-, (1→6)-α-glucans (e.g., dextran, glycogen, starch). Finally, depending on their constitution, they are indicated as homoglucans (only glucose molecules) or heteroglucans (not only glucose molecules) (19,20).

**Figure 1:**
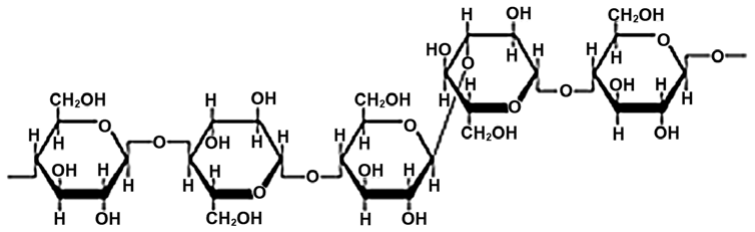
Example of (1→3)-β-D-glucan.

The β-glucans consist of linear unbranched polysaccharides of β-D-glucose. The basic β-D-glucan is a repeating structure with the β-D-glucose units joined together in linear chains by β-bonds. These can extend either from carbon 1 of one saccharide ring to carbon 3 of the next (β1→3) (Fig. 1), or from carbon 1 to carbon 4 (β1→4), or from carbon 1 to carbon 6 (β1→6) (1). The β-D-glucans can form large cylindrical molecules containing up to 250,000 glucose units.

As reported above, the sources of glucans are various, including fungi (e.g., mushrooms), yeast and seaweed, as well as barley. Medical glucans (as the ones used by traditional medicine) are principally obtained from edible fungi. By boiling and treating with enzymes from one of the cited sources, glucans can be extracted in crude form yielding soluble and insoluble products (19,21,22). There are many forms of soluble β-glucans evaluated for possible antitumor activity, such as (1→3)-β-D-glucan, SSG obtained from *Sclerotina sclerotiorum* IFO 9395 (23), SPG (also Schizophyllan, sizofiran, sonifilan) from *Schizophyllum commune*(24) and GRN (also Grifolan) from *Grifola frondosa*(25) and they often exist as a linear triple-helical structure in an aqueous solution (26). Insoluble glucans have been isolated for the first time from the mushroom *Lentinus edodes*(27). They were also isolated from the cell wall of yeast by using the combination of NaClO oxidation and dimethylsulfoxide (DMSO) extraction (28). To improve their solubility, derivatization by phosphorylation, either sulfation or amination can be used. However, insoluble β-glucans were found to possess higher immunostimulating activity than soluble ones and are administered orally. Factors that can greatly influence the antitumor and immunodulatory activities of the glucans are their structure, molecular weight, degree of branching and conformation (17,29–31). The molecular weight of glucans is dependent upon their source and extraction method (32). For example, the average molecular weight of Krestin (PSK), Lentinan, Schizophyllan (SPG) and PGG-glucan are, respectively, reported as 100,000, 500,000, 450,000 and 170,000 Da (33–35).

## 3. Immunostimulatory properties of glucans

As stated above, the immunostimulatory properties of fungal β-glucans were studied and described almost 50 years ago (36). Shortly afterwards, their effects against tumor development in experimental animals were also described (37) and finally glucans were reported to modulate other conditions (e.g., cholesterol levels, glucose tolerance) (38,39).

Since these early studies, it has been demonstrated that β-D-glucans increase the resistance of mammalians against several bacterial, fungal, viral and protozoal pathogens (40–43). A recent study compared the effects of soluble oat glucan versus Pleurotan, an insoluble β-D-glucan isolated from the mushroom *Pleurotus ostreatus*. They were administered as a food supplement for athletes and the β-D-glucan isolated from the mushroom resulted in significantly reducing the incidence of upper respiratory tract infection. Interestingly, the Pleurotan administration was associated with an increased number of circulating natural killer cells as well as a preventive effect on the reduction of natural killer cell activity. These latter findings may explain the reduced infectivity risk in the treated athletes (29). Since the soluble oat glucan supplementation did not produce effects on the incidence of respiratory tract infections, it was suggested that solubility and structural factors (e.g., backbone structure and degree of branching) can deeply affect the immunomodulatory capacity of β-D-glucans (17). Many studies have reported the ability of (1→3)-β-D-glucans to activate innate immunity with effects also on adaptive immunity, inducing humoral and cell-mediated immune responses. The (1→3)-β-D-glucans were found to increase the antimicrobial activity of mononuclear cells and neutrophils (7,44,45) and enhance the functional activity of macrophages (46,47). It has been reported that the (1–6)-branched type glucans, with high molecular weight and (1→3)-β-D-glucans are especially effective in inducing nitric oxide production by macrophages (21,47,48). Moreover, *ex vivo* experiments with macrophages obtained from animals treated with (1→3)-β-D-glucans showed enhanced esterase release and cytostatic effect on tumor cells when challenged with L-929 tumor cells (49). (1→3)-β-D-glucans were also reported to have hematopoietic activities, according to their conformation (single and triple helix) and to stimulate the proliferation of monocytes and macrophages (50–52). Relating to their role in triggering innate immunity responses, insoluble and derivatized (1→3)-β-D-glucans, according to their source, were also found to stimulate the production of proinflammatory molecules such as complement components, IL-1α/β, TNF-α, IL-2, IFN-γ and eicosanoids as well as IL-10, and IL-4 (53–59).

Protective effects of glucans were observed in mouse and rat models of sepsis (60–62). Neutrophils obtained from glucan-treated mice showed enhanced phagocytosis of *E. coli* in *ex vivo* experiments (63). *In vivo* administration of poly-[1-6]-β-D-glucopyranosyl-[1–3]-β-D-glucopyranose (PGG-glucan) in rats before bacterial challenge increased the number of leukocytes and also protected against lethal peritonitis (64). Similarly, in a mouse model of dental infection, PGG-glucan reduced infection-stimulated periapical bone resorption (65). The immunomodulatory properties of PGG-glucan studied also in many *in vitro* models evidenced that phagocytic cells (polymorphonuclear lymphocytes) increase their bactericidal capabilities when incubated in the presence of PGG-glucans. In purified human neutrophils, PGG-glucan was shown to induce the activation of an NFκB-like nuclear transcription factor. This activation was dependent on the binding of PGG-glucan to glycosphingolipid lactosylceramide expressed on the cell surface of neutrophyls (45). Berovic *et al* reported that one polysaccharide fraction isolated from *Ganoderma lucidum*, a mushroom rich in β-D-glucans, can induce TNF-α synthesis in primary cultures of human peripheral blood mononuclear cells (66). However, the protective effect of β-glucan against oxidative stress was also described using (1→3)-, (1→6)-β-D-glucan prepared from *Saccharomyces cerevisiae* yeast (62). These data support the observations of the ability of glucans to prevent and decrease infectious complications (53,67). Nevertheless, the various effects reported here indicate the necessity of a clear characterization of glucans by their origin, their structure and their fractions to better define the type of immune modulation elicited by each compound.

## 4. Glucan receptors on immune cells

The innate immunity cells are provided of a complex network of germ line-encoded pattern-recognition receptors (PRRs). They can identify pathogens by binding to carbohydrates, lipids and proteins expressed by the microorganism, including fungi (68–71). As reported above, *in vivo* administration of pure glucans induces the activation a wide range of responses by innate immunity (70,72). In particular, glucans have been found to react with one or multiple of the following cell surface receptors: complement receptor-3 (CR3), lactosylceramides, scavenger receptors and dectin-1 (73–76). Dectin-1 is considered the main β-D-glucan receptor. The β-D-glucan binding to myeloid cell receptors triggers, according to the bound receptor, a series of signaling events that modulate innate and subsequently adaptive immune responses, mainly through release of pro-inflammatory cytokines (IL-1α/β, IL-6, IL-8, IL-12, TNF-α) as well as cytotoxic molecules working also as inflammatory mediators [nitric oxide (NO) and hydrogen peroxide (H_2_O_2_)], as cited in the previous paragraph. The activation of macrophages performed by (1→3)-β-D-glucans is thought to be consequent to binding of the polymer to CR3 (CD11/CD18) receptors (42). The receptor-glucan interaction triggers phagocytosis, respiratory burst and secretion of cytokines such as TNF-α in addition to IL-10 (77,78). For an adequate use of glucans as immune enhancers, it is necessary to point out that glucan polymers derived from various sources can largely differ in binding affinity with specific receptors (from 24 *μ*M to 11 nM). Consequently, different biological effects can be promoted according to the source of the chosen molecule (68). Human monocytes (but also fibroblasts) express many glucan receptors which can differentiate between the polymers of (1→3)-β-D-glucan (68,79). Neutrophils exhibit lactosylceramide that mediates the response to PGG-glucan and CR3 mediates cytotoxicity for iC3b-opsonized target cells (35,80). CR3 receptor is also represented on natural killer cells (NK). Consequently, the triggering of complement alternative activation pathway by β-D-glucans with the availability of iC3b fragment elicits a high-avidity link of iC3b-opsonized cells (tumor cells or pathogens) to the receptors for iC3b and stimulates phagocytosis by monocytes and cytotoxic degranulation by NK cells (81). Macrophage/monocytes present on their surface scavenger receptors and dectin-1 recognizing (1→3)-β-D-glucans and non-opsonic zymosan. Dectin-1 is also represented on dendritic cells (see below) (82,83).

Some studies have suggested the complement receptor type 3 (CR3, also CD11b/CD18) is a prime candidate for β-D-glucan receptor on human monocytes, neutrophils and NK cells (80). More recently, dectin-1 was definitively identified as the most important β-D-glucan receptor (84). Human and murin dectin-1 mostly show a similar structure and function (85). Dectin-1 is a small type II transmembrane glycoprotein receptor containing one lectin-like carbohydrate recognition domain which is able to recognize (1→3)-β- and/or (1→6) β-D-glucans as well as fungi particles (86). This receptor is highly expressed on macrophages and granulocytes, but also on dendritic cells with effects on T and B cell responses (75,87,88). Dectin-1 presents two ligand-binding sites, one able to recognize the endogenous ligand on T cells and the other for exogenous carbohydrate (89). It has been shown that dectin-1 is able to mediate inflammatory cellular responses to β-D-glucans. The release of TNF-α, after interaction of β-D-glucans with the superficial part of the receptor, needs the cytoplasmic tail and immunoreceptor tyrosine activation motif of Dectin-1 as well as Toll-like receptor (TLR)-2 and Myd88 (71,73,90,91). The role of dectin-1 is important on dendritic cells (DCs) (73,75). Recent studies have shown the capability of DCs to stimulate antigen specific CD8^+^ T cell responses after dectin-1 is bound by the anti-dectin-1 antibody. The receptor-Ab interaction triggers a Syk-dependent pathway with upregulation of costimulatory molecules, secretion of cytokines and chemokines. This induces enhancement of antigen presentation, priming and expansion of antigen specific CD8^+^ T cells. A similar effect can be hypothesized after dectin-1 bounding to β-glucans (92).

Moreover, glucan-dependent dectin-1 signaling in macrophages and bone marrow-derived dendritic cells has been found to trigger the formation of LC3II, a central component in autophagy, as well as recruitment of LC3II to phagosomes. Here also Syk is involved. This promoted presentation of fungal-derived antigens to CD4 T cells occurs by facilitation of MHC class II molecule recruitment to phagosomes (93,94).

## 5. Antitumor activities of glucans

Polysaccharides from fruiting bodies, cultured mycelia and cultured filtrates of basidiomycetes have been reported to present antitumor activity. These antitumor polysaccharides are different in their chemical composition depending on their molecular weight, purity and degree of branches (3,82). As quoted by Bulmer *et al*(95), the first reports on the antitumor properties of extracts from fungi were published by Ringler in 1955 (a PhD thesis) and Lukas *et al*(96). Since then, many antitumor polysaccharides were isolated from fungi and extensively studied, especially in Japan (12,17,97–99). As has been emphasized, the therapeutic efficacy of these polysaccharides can greatly differ according to their chemical composition, configuration and physical properties. A wide range of glucans extending from homopolymers to highly complex heteropolymers were found to exhibit antitumor activity and most of the antitumor polysaccharides presented the same basic β-D-glucan structure with different types of glycosidic bounds. Glucans with high molecular weight appear to be more effective than those with low molecular weight (3,99). Differences in the effectiveness of mushroom glucan preparations are related to the type of polymer (according to the type of β-backbone) but also to the presence and proportion of various products in the same preparation. The simultaneous presence of different products may elicit multiple stimulatory activities with possible enhancement of the immunomodula-tory effects. A clear example of this possible collaboration, related to products obtained from *Agaricus blazei*, is reported by Borchers *et al* in their review on mushrooms as anticancer immune modulators (100). They assert that the mushroom *Agaricus blazei* contains more compounds [an antitumor glucan with a (1→6)-β-backbone, an (1→6)-α- and (1→4)-α-D-glucan complex and a glucomannan with a main chain of (1→2) β-linked D-mannopyranosyl residues] that were found to inhibit tumorigenesis (101–103). The preparation by aqueous extraction from powdered, dry fruiting body was less efficient than the direct administration of the complete dry powdered form. In rats fed with either aqueous extract or dry powdered preparation, the complete dry powder developed a better antimutagenic activity (104). Similar results were found also for diets containing powdered *Lentinula edodes* (shiitake) (105,106). The interpretation of Borchers *et al* is that different polysaccharides can cooperate by targeting different cell subsets by different receptors. Consequently, a more complex and effective stimulation would be more easily elicited when whole-mushroom extracts are used (100,102,107–109).

Polysaccharides or polysaccharide-protein complexes obtained from natural sources are generally reported to not produce direct cytotoxic action on tumor cells, but to induce host-mediated antitumor immune responses. However, the complete absence of direct effects on tumor cells cannot be totally excluded according to some recent studies (110–112). Pioneering studies of Di Luzio *et al*, using intravenous injection of soluble or particulate glucan, documented significant regressions of a syngeneic anaplastic mammary carcinoma and B16F10 melanoma in A/J and C57BL/6 mice, respectively (113). It has also been demonstrated that orally administrated yeast-derived as well as mushroom-derived β-(1–3) glucan had significant inhibitory effects on the growth of metastatic cancer cells using *in vivo* models of cancer (114,115). Animals that received treatment with PSK, β-(1→4)-D-glucans with (1→6)-β-glycopyranosidic side chains showed an increased number of neutrophils and a significant decrease in the size and number of lung metastasis (116). Therefore, the effects may not be limited only for use in the early stages of carcinogenesis or tumor development as suggested by the enhancement of immune responses (IL-1β, IFN-γ, TNF-α and IL-12 production, NK cell increase, macrophages activation), an increase of the host's antioxidant capacity and upregulation of phase I and phase II enzymes involved in the metabolic transformation as well as detoxification of mutagenic compounds (117,118). Finally, the efficacy of some types of fungal derivatives like lentinan, pachymaran, scleroglucan, curdland, grifolan and *Agaricus blazei* (1→3)-β-D-glucan resulted particularly high in various *in vivo* models of cancer. According to the reports, the tumor inhibition ratio in animal models range from 90.4 (scleroglucan) to 99.6% (lentinan) (119–122).

Glucans have also been proposed as an adjuvant. Some examples in animal models suggest an increasing of chemo- or immunotherapy efficacy when they are associated to polysaccharides, mainly glucans. The combination of an anti-MUC1 mAb with β-glucans significantly increased 20% the rate of RMA-S-MUC1 tumor regression in C57BL/6 mice (14). *Ganoderma lucidum* polysaccharides were also able to prolong the survival of Lewis carcinoma bearing C57BL/6 mice and to enhance antitumor activities of cytotoxic drugs and immunomodulators (123). Of particular interest is the possibility of using glucans for triggering complement-dependent antitumor cytotoxicity.

As previously cited, complement is a relevant mediator of antitumor β-D-glucan effects even after oral administration. Complement is an important part of the innate immunity against microorganisms that exhibit β-D-glucans as a surface component. These molecules are not expressed by tumor cells and, consequently, tumor cells cannot trigger CR3-dependent cellular cytotoxicity (CR3-DCC) (124). Oral administration of β-D-glucans may modify this condition. Glucan, in insoluble form, can be processed by gastrointestinal macrophages to soluble form. Once the soluble form is delivered, it can reach CR3 of bone marrow granulocytes and tissue macrophages making iC3b fragments available. In this way, the promotion of cytotoxity against tumor cells could be the result of contemporary presence of iC3b fragments and antitumor antibodies (125). Complement activation and deposition of iCR3 on tumor cells needs the presence of antitumor antibodies to produce a synergistic effect. Such an effect, leading to tumor regression, was evidenced by various authors using administration of β-D-glucans together with monoclonal antibodies against GD2 ganglioside, G250 protein, CD20 protein, respectively in experimental neuroblastoma, carcinoma and CD20^+^ lymphoma (126–128). Evidence of the dependence of this approach from complement involvement was given by failures of therapy in mice deficient in CR3 (CD11b^−/−^) or C3 (C3^−/−^) (129–131). This approach, since the progressively larger use of monoclonal antibodies in anticancer therapies, results in a particularly appealing and prospective application of β-D-glucans as effective enhancers of antitumor responses, as also demonstrated by recent literature (18,132).

## 6. Conclusion and potential

A substantial amount of literature has been accumulated in past decades on the medical potential of polysaccharides, particularly the β-D-glucans, from medical mushrooms used by the traditional medicine. Especially in recent years, the interest in these molecules or compounds has arisen together with the understanding of innate immunity implications during carcinogenesis and cancer development. Unfortunately, many clinical reports lack a specific rationale or simply describe effects according to traditional medicine application. However, some recent studies on gastric and colorectal cancer patients indicate the possible efficacy of these saccharides (133–135). Experimental studies have in large part clarified the basic mechanisms involved in the immune stimulation produced by β-D-glucans, especially with the knowledge on dectin-1 and C3-iCR3 involvement. A clear definition of the biologically active molecules and a more detailed chemical and biological characterization of the glucans from different sources appear necessary to better define the rationale of their application in anticancer therapies as well as other suitable pathologies. For example, it was suggested by Hamuro and Chihara that only extracts able to deactivate protein helices (as tested on bovine serum albumin) were active against tumors (136). Furthermore, β-D-glucans also appear suitable for use in nanomedicine for preparation of natural nanocarriers for drug or biological molecule delivery (137–139). The creation of gels or lattices based on β-D-glucans has also been proposed for various utilizations (e.g., in wound healing by stimulating macrophage activation and collagen deposition) (140,141). The addition of new areas of application, apart from the immunological use in oncology, opens new interesting perspectives and makes the study of β-D-glucan chemical and biological properties a prospective field of research.
